# Variation in Blood Pressure Classification Using 7 Blood Pressure Estimation Protocols Among Adults in Taiwan

**DOI:** 10.1001/jamanetworkopen.2020.24311

**Published:** 2020-11-18

**Authors:** Hung-Ju Lin, Heng-Yu Pan, Wen-Jone Chen, Tzung-Dau Wang

**Affiliations:** 1 Cardiovascular Center and Division of Cardiology, Department of Internal Medicine, National Taiwan University Hospital, Taipei, Taiwan; 2 Department of Internal Medicine, National Taiwan University Hospital Yun-Lin Branch, Douliu City, Taiwan

## Abstract

**Importance:**

Discrepancies in blood pressure (BP) estimates lead to incomparable BP assessment.

**Objective:**

To determine intraindividual discrepancies in BP estimates and classifications based on different BP estimation protocols.

**Design, Setting, and Participants:**

This cross-sectional study was a secondary analysis of data from the May Measurement Month Taiwan in 2017 and 2018, which were cross-sectional survey campaigns at pharmacies nationwide to raise awareness of high BP. Participants were volunteers aged 20 years or older. Analysis was conducted from February 2 to August 7, 2020.

**Exposure:**

Pharmacist-measured sitting BP using oscillometric sphygmomanometers.

**Main Outcomes and Measures:**

A total of 7 BP estimation protocols were assessed according to the latest American College of Cardiology (ACC), Chinese Hypertension League (CHL), European Society of Cardiology (ESC), International Society of Hypertension, Japanese Society of Hypertension, and National Institute of Health and Care Excellence (NICE) hypertension guidelines, and the proposed Averaging the Lowest Two systolic readings protocol. According to BP classification schemes of ESC and ACC guidelines, intraindividual discrepancies were identified if classification inconsistencies among 7 BP estimates were present.

**Results:**

Of 81 041 participants, 62 647 adults with 3 BP readings were included. The median (interquartile range) age was 59.0 (46.0-69.0) years, and 31 922 (51.5%) were women. The intraindividual maximum mean (SD) differences in systolic/diastolic BP estimates among the seven protocols were 4.8 (4.3)/3.3 (3.1) mm Hg. The highest prevalence of BP of 140/90 mm Hg or higher was by CHL (16 405 participants [26.2%]) and the lowest was by Averaging the Lowest Two (13 996 participants [22.3%]; *P* < .001); while the highest prevalence of 130/80 mm Hg or higher was by NICE (37 232 participants [59.4%]) and the lowest prevalence was by Averaging the Lowest Two (32 788 participants [52.4%]; *P* < .001). Compared with the other 6 estimates, Averaging the Lowest Two reclassified 7.3% to 15.8% of participants designated as 140/90 mm Hg or higher to less than 140/90 mm Hg, and 4.9% to 14.1% of those as 130/80 mm Hg or higher to less than 130/80 mm Hg. Intraindividual discrepancies in classifications occurred in 19 815 participants (31.6%) with the ESC classification and 16 401 participants (26.2%) with the ACC BP classification. Classification agreements were the lowest between NICE (κ coefficient, 0.667 [95% CI, 0.662-0.671]) and ESC protocols (κ coefficient, 0.705 [95% CI, 0.701-0.709]).

**Conclusions and Relevance:**

This cross-sectional study of adults in Taiwan found that different BP estimation protocols led to considerable intraindividual discrepancies in BP estimates and classifications. These findings suggest that the Averaging the Lowest Two protocol is less likely to overestimate BP and could serve as a prudent recommendation for BP estimation.

## Key Points

**Question:**

Are different blood pressure (BP) estimation protocols associated with discrepancies in BP estimates and classifications?

**Findings:**

In this cross-sectional study including 62 647 Taiwanese adults, discrepancies in BP classifications occurred in 31.6% and 26.2% of participants according to ESC and ACC classifications, respectively. The Averaging the Lowest Two protocol estimated the lowest prevalence of hypertension.

**Meaning:**

These findings suggest that a global consensus on BP estimation should be achieved to avoid incomparable BP assessment.

## Teaser

This cross-sectional study examines the intraindividaul discrepancies in blood pressure estimates and classifications based on different BP estimation protocols.

## Introduction

While increasing awareness of and screening for high blood pressure (BP) are important for improving BP control,^1^ obtaining a reliable BP estimate is the cornerstone for the BP-guided diagnosis and management of hypertension.^2^ Given that increasing visit-to-visit systolic BP variability by 5 mm Hg contributed to a 10% increase in the risk of death^3^ and lowering the definition of hypertension from 140/90 mm Hg or higher to 130/80 mm Hg or higher was associated with a 14% increase in prevalence,^4^ it is conceivable that variations of repeated BP measurements and inconsistent BP estimation protocols could lead to inaccurate assessment of cardiovascular risks and inappropriate management of hypertension.

BP varies with time and is subject to the effects of long-acting pathophysiological alterations superimposed by short-acting stress stimuli.^5^ However, the high reproducibility and low variations of BP measurements are fundamental to the reliability of BP estimates. While increased long-term BP variability is associated with higher cardiovascular risks,^3^ short-term BP variability, which compromises BP measurement reproducibility, could lead to differential performance of BP measurements derived from different clinical settings on cardiovascular risk predictions.^6^ To reduce the outcomes associated with of short-acting stress on the reproducibility of BP measurements, current hypertension guidelines unanimously provide standardized recommendations regarding how to accurately measure BP.^4,7,8^

On the contrary, current hypertension guidelines recommend different BP estimation protocols to derive BP estimates from 1 or more BP measurements.^8,9,10,11^ These BP estimation protocols differ in the strategies to deal with unstable BP measurements, such as whether the first BP reading of repeated measurements is included or not, because it seems to be more susceptible to stress stimuli and measurement errors than the subsequent readings.^12^ It remains unclear whether the intraindividual BP estimates and classifications are consistent based on different BP estimation protocols from current hypertension guidelines. Given that automated pharmacist-measured BP was similar to the widely recommended automated office BP,^13,14^ we analyzed data from individuals who underwent triplicate BP measurements by community pharmacists in May Measurement Month (MMM) Taiwan campaigns in 2017 and 2018 to investigate the discrepancies in and correlates of various BP estimates and classifications.

## Methods

The study protocols for this cross-sectional study were approved by the research ethics committee of National Taiwan University Hospital. All participants provided oral informed consent. The Strengthening the Reporting of Observational Studies in Epidemiology (STROBE) reporting guideline was followed to report this study.

### Study Design and Participants

We launched a cross-sectional, observational BP measurement study in May of 2017 and 2018, the MMM Taiwan campaign, which was affiliated with the International Society of Hypertension (ISH).^1,10,15^ Because the campaign was aimed at raising the awareness of high BP in the general population, we used community pharmacies as BP screening sites and enrolled a total of 81 041 adults aged 20 years or older to join this campaign. Participants were asked to fill out a structured questionnaire regarding medical history of diabetes, coronary heart disease, stroke, hypertension, lifestyle habits of smoking and alcohol consumption, and frequency of practicing home BP measurement in the preceding year (eTable 1 in the Supplement).

### Triplicate BP Measurements and BP Variability Patterns

In the campaigns, community pharmacists followed the standardized procedure to take triplicate BP measurements of participants using calibrated automated oscillometric sphygmomanometers. The cuff size was determined according to the arm circumference of participants and the BP device manufacturer’s recommendations. After participants took a 5-minute sitting rest, 3 consecutive BP readings of the right or left arm were taken in a proper sitting position by community pharmacists, spacing each BP measurement at least 1 minute apart. The BP variability patterns of the triplicate BP measurements were categorized into 1 of 3 groups according to the sequential changes of the triplicate SBP readings: the descending group if the triplicate SBP readings were in descending order; the ascending group if the triplicate SBP readings were in ascending order; or otherwise, the fluctuating group.

### BP Estimation Protocols

BP estimation protocols are approaches to derive BP estimates from 1 or more BP measurements. The key differences between the protocols from various hypertension guidelines lie on the strategies to determine and manage the potentially biased BP measurements. These strategies include 2 sectors: whether selection criteria, like the diagnostic threshold of hypertension or a threshold of significant BP difference between consecutive measurements, for BP measurements are applied and how the means of BP measurements were obtained (ie, calculating the mean from all, calculating the mean from the last 2, calculating the mean from the 2 with the least difference, calculating the mean from the lowest 2, and picking the lowest 1). In this study, we compared the 7 BP estimation protocols. Of these protocols, 6 were suggested by the latest American College of Cardiology (ACC),^4^ Chinese Hypertension League (CHL),^10^ European Society of Cardiology (ESC),^8^ ISH,^13^ Japanese Society of Hypertension (JSH),^9^ and National Institute of Health and Care Excellence (NICE)^16^ hypertension guidelines. Given the unpredictable short-term BP variability despite 5-minute rest and the occasional unparalleled variations between systolic BP and diastolic BP, we proposed the Averaging the Lowest Two protocol, with which the BP estimate was calculated as the mean from the 2 BP measurements with the lowest systolic BP readings. The reasons for specifying systolic BP readings in our protocol are first, to avoid confusion when there are inconsistencies in systolic BP and diastolic BP with regard to the selection criterion, and second, systolic BP are in general more prognostically significant than diastolic BP (eAppendix and eFigure in the Supplement).^17,18^

### Statistical Analysis

To derive the 7 BP estimates for each individual, we analyzed data from 62 647 participants with complete records of triplicate BP measurements. To explore the potential selection bias, inverse probability weighting–adjusted comparisons of the systolic BP and diastolic BP means were made between individuals with and without complete records of triplicate BP readings (eAppendix in the Supplement). Continuous variables were presented as means with SDs, and categorical variables were presented as numbers with percentages of nonmissing data.

To evaluate agreements and discrepancies in BP classifications, the 2 distinctive BP classification schemes were used according to the latest ESC and ACC guidelines.^4,8^ The ESC BP classification scheme consists of 6 BP grades, including optimal (<120/80 mm Hg), normal (120-129/80-84 mm Hg), high normal (130-139/85-89 mm Hg), grade 1 hypertension (140-159/90-99 mm Hg), grade 2 hypertension (160-179/100-109 mm Hg), and grade 3 hypertension (≥180/≥110 mm Hg).^8^ The ACC BP classification scheme consists of four BP categories, including normal (<120/80 mm Hg), elevated (120-129/<80 mm Hg), stage 1 hypertension (130-139/80-89 mm Hg), and stage 2 hypertension (≥140/≥90 mm Hg).^4^

The intraindividual BP estimates and differences of systolic BP pairs were considered as correlated variables for comparisons. The Cochran-Mantel-Haenszel method was used to assess discrepancies in BP classifications across the 7 BP estimates. The Fleiss κ coefficient was used to assess the overall level of agreement among the 7 BP estimation protocols; while the Cohen κ coefficient was used to assess the agreement between any 2 protocols according to the ESC and ACC BP classification schemes. The level of agreement was considered acceptable if κ coefficient of 0.8 or greater. The pairwise comparisons of the dependent κ coefficients were made using the Hotelling T square test with the variance-covariance matrix constructed by 1000 bootstraps.^19^

A multivariable logistic regression model in which the means of triplicate systolic BP and diastolic BP were adjusted was used to explore whether the clinical features and BP variability patterns were related to the discrepant BP classifications among the 7 BP estimates. Discrepant BP classifications were defined as the presence of any intraindividual inconsistency in the ACC or ESC BP classifications by the 7 BP estimates. The variables fitted into the multivariable model included age, body mass index, sex, medical history of coronary artery disease, diabetes, and hypertension, current smoker, alcohol consumption, frequency of home BP monitoring, arm of BP measurement, and BP variability patterns (eAppendix in the Supplement).

The 2-sided *P* < .05 was considered statistically significant. The statistical analysis was performed using the SAS software version 9.4. (SAS Institute), and R software version 3.6.1. (R Project for Statistical Computing). Analysis was conducted from February 2, 2020, to August 7, 2020.

## Results

Of 62 647 participants with a median (interquartile range) age of 59.0 (46.0-69.0) years, 31 922 (51.5%) were women (Table 1). A total of 18 628 participants (31.5%) took BP measurements at home once or more weekly in the preceding year, while 18 086 participants (30.6%) did not take any home BP measurement in the preceding year before joining the campaigns. Compared with participants with triplicate BP readings, participants without triplicate BP readings had lower systolic BP (mean difference, −1.0 [95% CI, −1.5 to −0.6] mm Hg; *P* < .001) and similar diastolic BP (mean difference, 0.1 [95% CI, −0.4 to 0.2] mm Hg; *P* = .69) (eTable 2 in the Supplement).

**Table 1.** Clinical Features and Blood Pressure Measures Among Participants of the Community-Based May Measurement Month Taiwan Campaigns

Characteristic	Total, No. (%) (N = 62 647)
Age, y	57.1 (15.9)
<50.0	22 376 (35.7)
50.0-59.9	11 574 (18.5)
60.0-69.9	15 127 (24.1)
≥70	13 570 (21.7)
BMI	24.4 (3.7)
<24.0	31 232 (49.9)
24.0-26.9	18 182 (29.0)
≥27.0	13 233 (21.1)
Sex	
Women	31 922 (51.5)
Men	30 078 (48.5)
Medical history	
Stroke	1765 (2.9)
Coronary artery disease	6436 (10.4)
Diabetes	12 964 (21.2)
Hypertension	28 096 (44.9)
Lifestyle habits	
Current smoker	10 818 (17.4)
Alcohol consumption	7904 (12.8)
Frequency of home BP monitoring, d/wk	
Never	18 086 (30.6)
<1	22 447 (37.9)
1-3	9590 (16.2)
4-6	3394 (5.7)
Daily	5644 (9.6)
Arm of taking BP measurements	
Right	23 682 (41.3)
Left	33 731 (58.7)
**Triplicate BP measurements**	
Systolic, mm Hg	
First	128.4 (17.9)
Second	126.6 (17.2)
Third	125.6 (16.9)
Diastolic, mm Hg	
First	78.8 (12.1)
Second	77.9 (11.7)
Third	77.3 (11.5)
Pulse rate, beats per min	
First	77.4 (10.9)
Second	76.6 (10.4)
Third	76.3 (10.2)
**Mean of repeated BP measurements**	
Systolic, mm Hg	
First and second	127.5 (17.2)
Second and third	126.1 (16.8)
All	126.9 (16.8)
Diastolic, mm Hg	
First and second	78.4 (11.6)
Second and third	77.6 (11.3)
All	78.0 (11.3)
Variability patterns of triplicate Systolic BP measurements	
Descending	18 363 (29.3)
Fluctuating	38 892 (62.1)
Ascending	5392 (8.6)
Absolute differences between BP measurements	
First and second systolic BP readings, mm Hg	5.3 (5.6)
Difference >5 mm Hg	20 042 (32.0)
Difference >10 mm Hg	7268 (11.6)
Second and third systolic BP readings, mm Hg	4.4 (4.9)
Difference >5 mm Hg	16 485 (26.3)
Difference >10 mm Hg	5176 (8.3)
First and second diastolic BP readings, mmHg	3.7 (4.3)
Difference >5 mm Hg	11 599 (18.5)
Difference >10 mm Hg	3183 (5.1)
Second and third diastolic BP readings, mm Hg	3.2 (3.7)
Difference >5 mm Hg	9350 (14.9)
Difference >10 mm Hg	2280 (3.6)
**BP estimation protocols**	
Systolic, mean (SD), mm Hg	
ACC protocol	126.9 (16.8)
CHL protocol	127.1 (16.9)
ESC protocol	126.1 (16.8)
ISH protocol	126.3 (16.5)
JSH protocol	126.7 (17.0)
NICE protocol	126.7 (16.3)
Averaging the Lowest Two protocol	124.9 (16.8)
Diastolic, mean (SD), mm Hg	
ACC protocol	78.0 (11.3)
CHL protocol	78.2 (11.4)
ESC protocol	77.6 (11.3)
ISH protocol	77.7 (11.3)
JSH protocol	78.0 (11.4)
NICE protocol	78.0 (11.4)
Averaging the Lowest Two protocol	77.3 (11.3)

Abbreviations: ACC, American College of Cardiology; BMI, body mass index (calculated as weight in kilograms divided by height in meters squared); BP, blood pressure; CHL, Chinese Hypertension League; ESC, European Society of Cardiology; ISH, International Society of Hypertension; JSH, Japanese Society of Hypertension; NICE, National Institute of Health and Care Excellence.

### The Triplicate BP Measurements

Although the overall means of the first, second, and third systolic BP and diastolic BP readings were both in decreasing order, the BP variability of triplicate BP measurements was categorized as the descending pattern in 18 363 participants (29.3%); the ascending pattern in 5392 participants (8.6%); and the fluctuating pattern in 38 892 participants (62.1%). Of the triplicate systolic BP readings, the highest was the first in 27 679 participants (54.1%); the second in 12 543 participants (24.5%), and the third in 10 952 participants (21.4%), after excluding measurements with identical SBP readings. There were 19 838 participants (31.7%) who had a first BP measurement of 140/90 mm Hg or greater. Given that BP measurements with the smallest difference between BP readings were regarded as stable, it is noteworthy that the lowest 2 systolic BP readings were more likely to have the smallest in-between systolic BP and diastolic BP differences than the other 2 systolic BP pairs (eTable 3 in the Supplement).

### Discrepancies Among the BP Estimates and Classifications

The mean (SD) for the intraindividual maximum differences in BP estimates among the 7 BP estimation protocols were 4.8 (4.3) mm Hg for systolic BP and 3.3 (3.1) mm Hg for diastolic BP. Of the 7 BP estimates, the CHL protocol had the highest the mean (SD) estimates for systolic BP (127.1 [16.9] mm Hg; *P* < .001) and diastolic BP (78.2 [11.4] mm Hg; *P* < .001) (Table 1). In the descending BP variability group, the mean systolic BP estimate derived from the NICE protocol was the highest, while those derived from the ESC and the Averaging the Lowest Two protocols were the lowest (eTable 4 in the Supplement). In contrast, the mean SBP estimate derived from the ESC protocol was the highest and that from the NICE protocol was the lowest in the ascending BP variability group (eTable 4 in the Supplement).

There were significant discrepancies in BP classifications among the 7 protocols according to the ESC and the ACC classification schemes (Table 2). The discrepancies in high BP classifications appeared to increase when the cutoff BP was lowered from 140/90 mm Hg to 130/80 mm Hg. The greatest difference in the prevalence of participants with BP estimates of 140/90 mm Hg or greater was 3.9 percentage points between the CHL and Averaging the Lowest Two protocols (16 405 participants [26.2%] vs 13 996 participants [22.3%]; *P* < .001). The greatest difference in the prevalence of participants with BP estimates of 130/80 mm Hg or higher was 7.0 percentage points between the NICE and Averaging the Lowest Two protocols (37 232 participants [59.4%] vs 32 788 participants [52.4%]; *P* < .001).

**Table 2.** Comparisons of Distributions of ESC and the ACC Blood Pressure Classifications According to Estimates From 7 Blood Pressure Estimation Protocols

Classification	Estimation protocol, No. (%) (N = 62 647)							*P* value
	ACC	CHL	ESC	ISH	JSH	NICE	ALT	
ESC								
Optimal	18 517 (29.6)	18 248 (29.1)	19 145 (30.6)	17 264 (27.6)	18 665 (29.8)	16 805 (26.8)	20 757 (33.1)	<.001
Normal	14 170 (22.6)	13 972 (22.3)	14 390 (23.0)	17 916 (28.6)	13 954 (22.3)	13 578 (21.7)	14 336 (22.9)	
High normal	14 114 (22.5)	14 022 (22.4)	14 043 (22.4)	12 599 (20.1)	13 995 (22.3)	17 529 (28.0)	13 558 (21.7)	
Grade 1 hypertension	12 472 (19.9)	12 920 (20.6)	11 905 (19.0)	11 717 (18.7)	12 609 (20.1)	11 542 (18.4)	11 106 (17.7)	
Grade 2 hypertension	2576 (4.1)	2668 (4.3)	2408 (3.8)	2400 (3.8)	2616 (4.2)	2400 (3.8)	2212 (3.5)	
Grade 3 hypertension	798 (1.3)	817 (1.3)	756 (1.2)	751 (1.2)	808 (1.3)	793 (1.3)	678 (1.1)	
ACC								
Normal	18 517 (29.6)	18 248 (29.1)	19 145 (30.6)	17 264 (27.6)	18 665 (29.8)	16 805 (26.8)	20 757 (33.1)	<.001
Elevated	9229 (14.7)	9040 (14.4)	9371 (15.0)	11 080 (17.7)	9026 (14.4)	8610 (13.8)	9102 (14.5)	
Stage 1 hypertension	19 055 (30.4)	18 954 (30.3)	19 062 (30.4)	19 435 (31.0)	18 923 (30.2)	22 497 (35.9)	18 792 (30.0)	
Stage 2 hypertension	15 846 (25.3)	16 405 (26.2)	15 069 (24.0)	14 868 (23.7)	16 033 (25.6)	14 735 (23.5)	13 996 (22.4)	

Abbreviations: ACC, American College of Cardiology; ALT, Averaging the Lowest Two systolic readings; CHL, Chinese Hypertension League; ESC, European Society of Cardiology; ISH, International Society of Hypertension; JSH, Japanese Society of Hypertension; NICE, National Institute of Health and Care Excellence.

**Figure 1.**
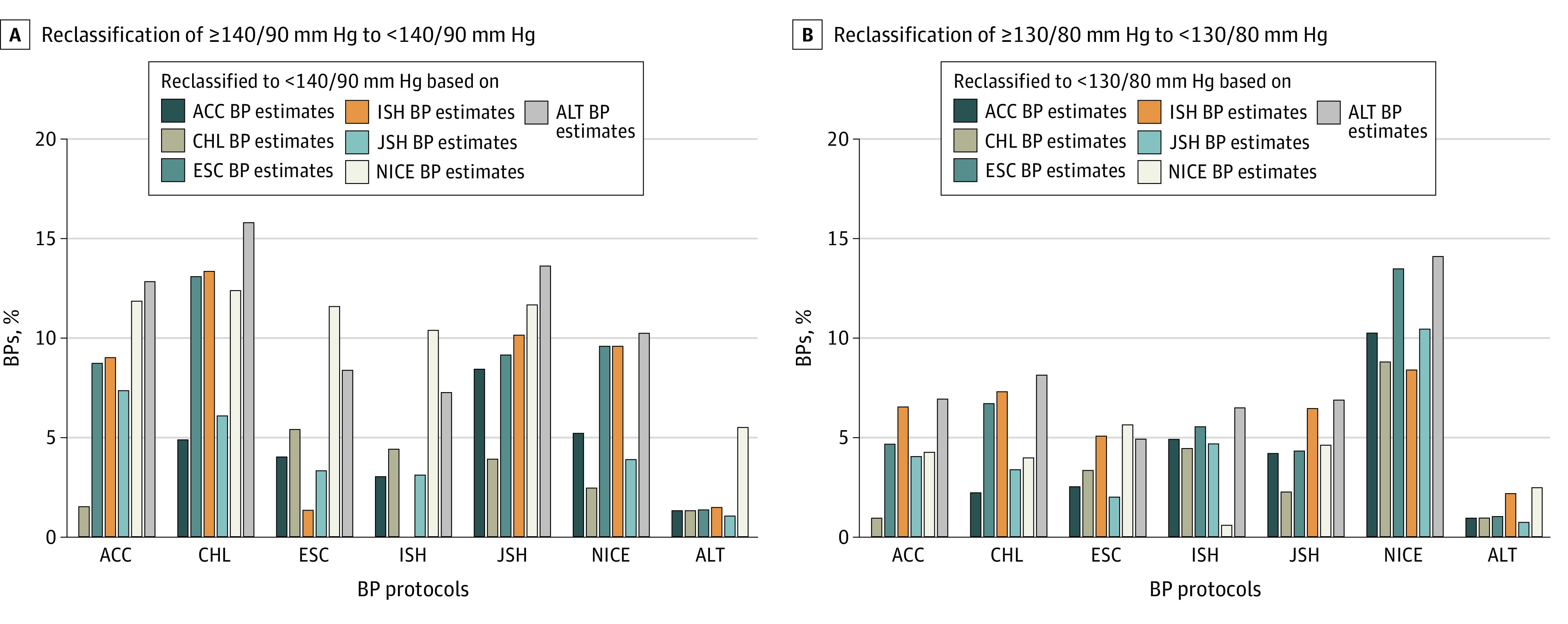
Reclassification of High Blood Pressures (BPs) Among Different Blood Pressure Estimation Protocols The reclassification proportions of high BP, (A) 140/90 mm Hg or greater to less than 140/90 mm Hg or (B) 130/80 mm Hg or greater to less than 130/80 mm Hg classification between the BP estimation protocols of the latest American College of Cardiology (ACC), Chinese Hypertension League (CHL), European Society of Cardiology (ESC), International Society of Hypertension (ISH), Japanese Society of Hypertension (JSH), and National Institute of Health and Care Excellence (NICE) hypertension guidelines, and the proposed Averaging the Lowest Two (ALT) protocol.

Compared with other estimates, the Averaging the Lowest Two estimates reclassified the largest proportions of individuals with high BP classifications based on other protocols, except the ESC protocol, into the normotensive classification (Figure 1). The Averaging the Lowest Two protocol reclassified the largest proportions of participants designated as 140/90 mm Hg or greater to less than 140/90 mm Hg in the CHL (2592 participants [15.8%]), JSH (2183 participants [13.6%]), ACC (2033 participants [12.8%]), and NICE (1509 participants [10.2%]) protocols, and second largest in the ESC (1262 participants [8.4%]) and ISH (1078 participants [7.3%]) protocols (*P* < .001) (Figure 1A). The Averaging the Lowest Two protocol reclassified the largest proportions of participants designated as 130/80 mm Hg or greater to less than 130/80 mm Hg in the NICE (5253 participants [14.1%]), CHL (2879 participants [8.1%]), JSH (2405 participants [6.9%]), ACC (2419 participants [6.9%]), and ISH (2225 participants [6.5%]) protocols, and third largest in the ESC protocol (1675 participants [4.9%]; *P* < .001) (Figure 1B).

The overall agreement (SE) of BP classifications assigned by BP estimates according to the 7 BP estimation protocols was 0.809 (0.0005) for the ESC BP classification scheme and 0.830 (0.001) for the ACC BP classification scheme, both of which were considered acceptable. However, the pairwise agreements between BP classifications by the 7 BP estimates varied greatly. For the ESC classification scheme, the NICE protocol had the lowest levels of agreement with the other protocols, except with the ISH protocol (Table 3). Similarly, for the ACC classification scheme, the NICE protocol had the lowest levels of agreement with the other protocols, except with the CHL and ISH protocols.

**Table 3.** Agreements in BP Classifications Using Pairwise Comparisons Between 7 BP Estimation Protocols According to the ESC and the ACC BP Classification Schemes

Protocol	Estimation protocol					
	CHL	ESC	ISH	JSH	NICE	ALT
ACC						
Consistent ESC BP classifications, %	59 290 (94.6)	55 377 (88.4)	53 427 (85.3)	53 583 (85.5)	49 852 (79.6)	54 498 (87.0)
Coefficient of agreement, κ (95% CI)	0.930 (0.928-0.933)	0.849 (0.846-0.852)	0.809 (0.805-0.812)	0.812 (0.808-0.816)	0.735 (0.731-0.739)	0.830 (0.827-0.833)
CHL						
Consistent ESC BP classifications, %	NA	52 253 (83.4)	51 876 (82.8)	56 446 (90.1)	51 338 (81.9)	53 087 (84.7)
Coefficient of agreement, κ (95% CI)	NA	0.784 (0.780-0.788)	0.777 (0.773-0.781)	0.872 (0.868-0.875)	0.766 (0.762-0.770)	0.801 (0.797-0.804)
ESC						
Consistent ESC BP classifications, %	NA	NA	56 014 (89.4)	56 101 (89.5)	46 587 (74.4)	57 178 (91.3)
Coefficient of agreement, κ (95% CI)	NA	NA	0.862 0.859-0.865)	0.864 (0.861-0.867)	0.667 (0.662-0.671)	0.886 (0.883-0.888)
ISH						
Consistent ESC BP classifications, %	NA	NA	NA	53 007 (84.6)	53 220 (84.9)	55 425 (88.5)
Coefficient of agreement, κ (95% CI)	NA	NA	NA	0.800 (0.797-0.804)	0.805 (0.801-0.809)	0.849 (0.846-0.853)
JSH						
Consistent ESC BP classifications, %	NA	NA	NA	NA	49 864 (79.6)	54 851 (87.6)
Coefficient of agreement, κ (95% CI)	NA	NA	NA	NA	0.735 (0.731-0.739)	0.837 (0.834-0.841)
NICE						
Consistent ESC BP classifications, %	NA	NA	NA	NA	NA	48 842 (78.0)
Coefficient of agreement, κ (95% CI)	NA	NA	NA	NA	NA	0.713 (0.708-0.717)
ACC						
Consistent ACC BP classifications, %	59 846 (95.5)	56 637 (90.4)	53 976 (86.2)	55 301 (88.3)	52 131 (83.2)	55 896 (89.2)
Coefficient of agreement, κ (95% CI)	0.939 (0.937-0.941)	0.869 0.866-0.873)	0.813 (0.809-0.816)	0.840 (0.837-0.844)	0.771 (0.767-0.775)	0.853 (0.850-0.856)
CHL						
Consistent ACC BP classifications, %	NA	54 054 (86.3)	53 204 (84.9)	57 656 (92.0)	53 324 (85.1)	54 733 (87.4)
Coefficient of agreement, κ (95% CI)	NA	0.813 (0.810-0.817)	0.796 (0.792-0.800)	0.892 (0.889-0.894)	0.797 (0.793-0.800)	0.828 (0.824-0.831)
ESC						
Consistent ACC BP classifications, %	NA	NA	55 335 (88.5)	57 273 (91.4)	49 126 (78.4)	58 019 (92.6)
Coefficient of agreement, κ (95% CI)	NA	NA	0.844 (0.841-0.848)	0.883 (0.880-0.886)	0.705 (0.701-0.709)	0.899 (0.896-0.902)
ISH						
Consistent ACC BP classifications, %	NA	NA	NA	53 922 (86.1)	56 328 (89.9)	55 423 (88.5)
Coefficient of agreement, κ (95% CI)	NA	NA	NA	0.811 (0.808-0.815)	0.863 (0.860-0.866)	0.844 (0.840-0.847)
JSH						
Consistent ACC BP classifications, %	NA	NA	NA	NA	51 918 (82.9)	56 196 (89.7)
Coefficient of agreement, κ (95% CI)	NA	NA	NA	NA	0.766 (0.762-0.770)	0.859 (0.856-0.863)
NICE						
Consistent ACC BP classifications, %	NA	NA	NA	NA	NA	51 252 (81.8)
Coefficient of agreement, κ (95% CI)	NA	NA	NA	NA	NA	0.751 (0.747-0.755)

Abbreviations: ACC, American College of Cardiology; ALT, Averaging the Lowest Two; BP, blood pressure; CHL, Chinese Hypertension League; ESC, European Society of Cardiology; ISH, International Society of Hypertension; JSH, Japanese Society of Hypertension; NA, not applicable; NICE, National Institute of Health and Care Excellence.

### Intraindividual Discrepancies in BP Classifications Associated With Clinical Features and BP Variability Patterns

**Figure 2.**
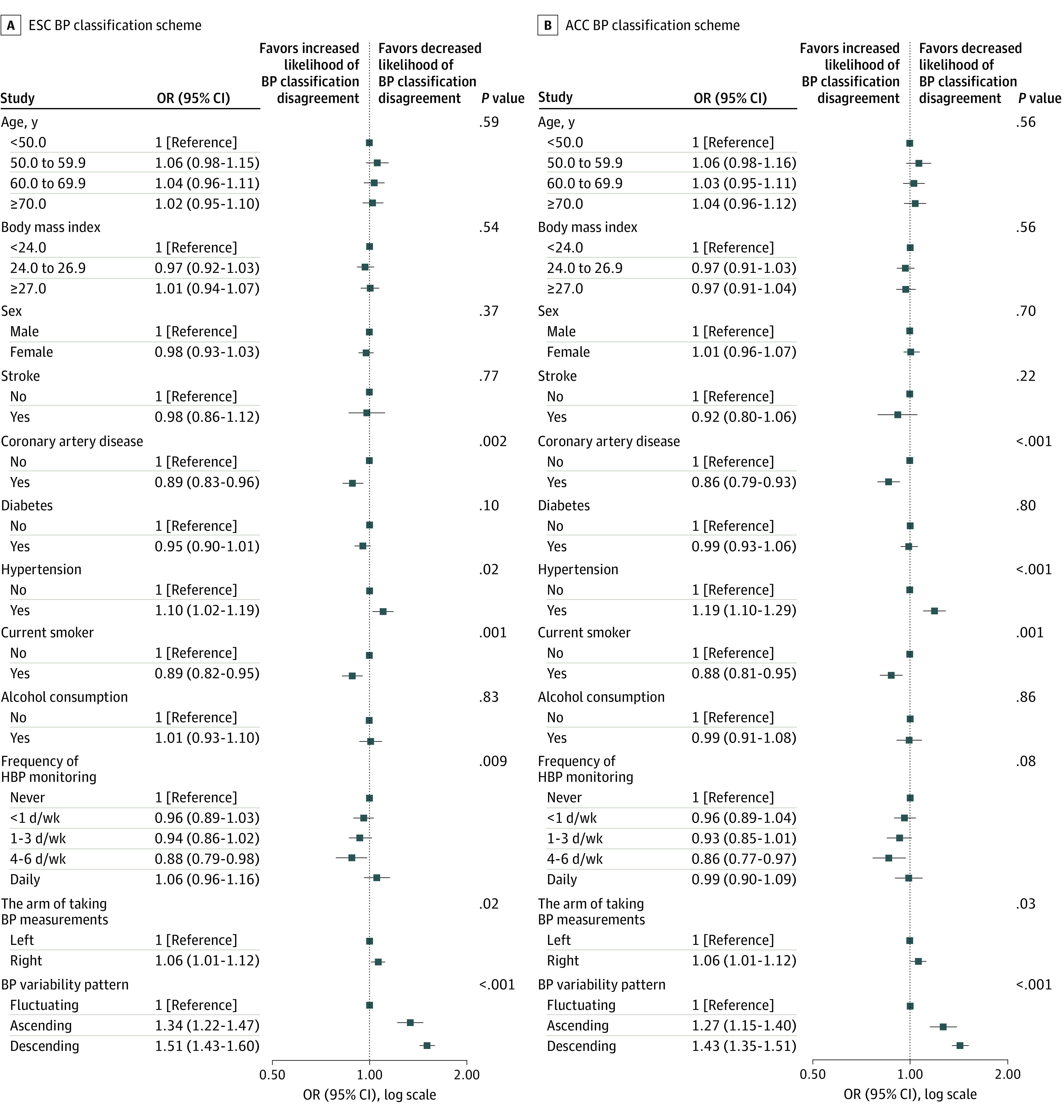
Clinical Covariates in Association With Intraindividual Discrepancy in Blood Pressure (BP) Classifications Among Different Estimation Protocols Multivariable-adjusted relationships of clinical features and BP variability patterns with the discrepant BP classifications among different BP estimates derived from the 7 BP estimation protocols. ESC indicates European Society of Cardiology; ACC, American College of Cardiology; OR, odds ratios; and HBP, home BP.

The intraindividual inconsistencies in BP classifications according to the 7 BP estimates, occurred in 19 815 participants (31.6%) with ESC classification and 16 401 participants (26.2%) with ACC classification. With the ESC classification scheme, the intraindividual discrepant BP classifications of the 7 BP estimates were more pronounced in individuals with hypertension, and those taking right arm BP and less likely in those with coronary artery disease and current smokers (Figure 2A). Except for participants performing daily home BP monitoring, intraindividual BP classification discrepancy tended to be less with increasing frequency of home BP monitoring. Participants with the ascending or descending patterns were more likely to have BP classification discrepancies, compared with participants with the fluctuating pattern. Outcomes were comparable when the ACC BP classification scheme was applied, except for home BP monitoring (Figure 2B).

## Discussion

This cross-sectional study found that approximately 30% of the triplicate BP measurements were manifested as the descending BP variability pattern and that the first systolic BP reading was the highest in only half of all participants. Among the 7 BP estimation protocols, the Averaging the Lowest Two protocol was associated with the lowest prevalence of hypertension, lower BP estimates across the BP variability groups, and higher rates of reclassification from hypertension to nonhypertension, indicating that the Averaging the Lowest Two protocol had a reduced tendency for BP overestimation. The BP variability patterns of triplicate BP measurements, together with the presence of hypertension and coronary artery disease, current smokers, and the right arm BP measurement, were significantly associated with the intraindividual discrepant BP classifications of the 7 BP estimates.

### Discrepancies Among the BP Estimation Protocols

The BP estimation protocols are designed to obtain the BP estimates from 1 or more BP measurements,^4,8,9,10,16^ of which the potentially biased BP measurements are trimmed or weighted to reduce the variations of BP estimates.^20^ Given the uncertainty of individual BP distributions, current strategies to determine and manage the potentially biased BP measurements are 3-fold. The first strategy, such as the ESC and ISH protocols,^8^ is to discard the first BP measurement, which often, but not necessarily, shows greater deviation from the following BP measurements.^8^ The second strategy is to minimize the impact of considerable differences between consecutive BP measurements by either calculating the mean of the consecutive 2 measurements with the minimal difference or calculating the mean of all 3 BP measurements taken on 1 occasion, such as the JSH, CHL, and ACC protocols.^9,10^ The third strategy is to choose only the lowest reading to avoid bias induced by short-acting stress, such as the NICE protocol.^16^ However, the NICE protocol was designed on the premise that the definition of hypertension is 140/90 mm Hg or greater, which made it overestimate the number of individuals with hypertension when hypertension is defined as 130/80 mm Hg or greater, as in this study. In other words, only 1 BP measurement, as recommended in the NICE protocol if it is less than 140/90 mm Hg, might not be viewed as an accurate BP estimate, as BP variations still occur no matter how low the BP level is. In this study, 8.6% of individuals had an ascending variability pattern of triplicate BP measurements, which implies that short-acting stress might manifest as not only the highest first BP reading in some individuals, but also the continuously increasing BP values of the triplicate measurements in other individuals. While the Averaging the Lowest Two protocol is a systolic BP–oriented strategy taking the short-acting impact of external or internal stress and its varied presentation into consideration,^21^ our findings showed that the lowest 2 systolic BP readings, not necessarily consecutive as requested in the JSH protocol, were more likely to have the lowest in-between systolic BP and diastolic BP differences.

### Limitations

Our study has some limitations. First, the MMM Taiwan campaigns were carried out solely in community pharmacies owing to limited logistic support. Therefore, the pharmacist-measured BP readings and the proportions of BP variability patterns could not be compared with those from other BP measurement types, such as automated office BP, which is considered a reliable reference standard of BP measurement.^13^ A prior randomized study has shown that automated office BP was similar to automated BP taken by pharmacists,^14^ whose role in controlling cardiovascular risk factors is emerging.^22^ Accordingly, our analyses of automated pharmacy BP could provide insights in determining the variations associated with different BP estimation protocols designed for automated office BP measurements. Second, given that there was difference in systolic BP between individuals with and without triplicate BP readings, the generalizability of our findings might be limited by this potential selection bias.

## Conclusions

The findings of this cross-sectional study of adults in Taiwan extend prior observations.^23,24,25^ To our knowledge, this is the first study to demonstrate that there are considerable differences in BP estimates and classifications among different BP estimation protocols from the contemporary hypertension guidelines, probably leading to improper BP management. Compared with protocols from different guidelines, BP estimates obtained by calculating the mean of the 2 BP measurements with the lowest systolic BP readings from triplicate BP measurements, the Averaging the Lowest Two protocol, are more likely derived from relatively stable BP measurements, and less tended to overestimate BP classifications. The Averaging the Lowest Two protocol could serve as a prudent recommendation for BP estimation, especially when lower BP targets are considered.

## Notes

Supplement. **eAppendix.** Supplemental Methods **eFigure.** Flowcharts Depicting the Blood Pressure Estimation Protocols **eTable 1.** Questionnaire Used for Collecting Clinical Features, Lifestyle Habits, and Blood Pressure Records **eTable 2.** Baseline Characteristics of Participants With and Without Complete Records of Triplicate BP Readings **eTable 3.** Comparison of the Absolute Differences Between Systolic Blood Pressure and Diastolic Blood Pressure Readings of the Triplicate Measurements Compared With Pairs of the Lowest 2 Systolic Blood Pressure Readings **eTable 4.** Clinical Features and Blood Pressure Measures Among Participants Stratified by Variability Patterns of Triplicate Blood Pressure Measurements **eReferences**
