# Supplementary material for: Variation in Blood Pressure Classification Using 7 Blood Pressure Estimation Protocols Among Adults in Taiwan
Source: JAMA Netw Open. 2020 Nov 18;3(11):e2024311. doi: 10.1001/jamanetworkopen.2020.24311 (PMC7675105; doi:10.1001/jamanetworkopen.2020.24311)
Supplement: Supplement. — eAppendix. Supplemental Methods eFigure. Flowcharts Depicting the Blood Pressure Estimation Protocols eTable 1. Questionnaire Used for Collecting Clinical Features, Lifestyle Habits, and Blood Pressure Records eTable 2. Baseline Characteristics of Participants With and Without Complete Records of Triplicate BP Readings eTable 3. Comparison of the Absolute Differences Between Systolic Blood Pressure and Diastolic Blood Pressure Readings of the Triplicate Measurements Compared With Pairs of the Lowest 2 Systolic Blood Pressure Readings eTable 4. Clinical Features and Blood Pressure Measures Among Participants Stratified by Variability Patterns of Triplicate Blood Pressure Measurements eReferences [file jamanetwopen-e2024311-s001.pdf]

Supplemental Online Content

Lin HJ, Pan HY, Chen WJ, Wang TD. Variation in blood pressure classification using 7 blood pressure estimation protocols among adults in Taiwan. *JAMA Netw Open*. 2020;3(11):e2024311. doi:10.1001/jamanetworkopen.2020.24311

eAppendix. Supplemental Methods
eFigure. Flowcharts Depicting the Blood Pressure Estimation Protocols
eTable 1. Questionnaire Used for Collecting Clinical Features, Lifestyle Habits, and Blood Pressure Records
eTable 2. Baseline Characteristics of Participants With and Without Complete Records of Triplicate BP Readings
eTable 3. Comparison of the Absolute Differences Between Systolic Blood Pressure and Diastolic Blood Pressure Readings of the Triplicate Measurements Compared With Pairs of the Lowest 2 Systolic Blood Pressure Readings
eTable 4. Clinical Features and Blood Pressure Measures Among Participants Stratified by Variability Patterns of Triplicate Blood Pressure Measurements
eReferences

This supplemental material has been provided by the authors to give readers additional information about their work.

© 2020 Lin HJ et al. *JAMA Network Open.*

eAppendix. Supplemental Methods

BP estimation protocols

We compared the seven BP estimation protocols, including the six protocols of the latest hypertension guidelines and the one we proposed, which were depicted in the **eFigure 1** in the **Supplement**. The first was the protocol of the American College of Cardiology (ACC) guideline,[1] which recommended that the BP estimate was the average of all triplicate BP measurements (**eFigure 1A** in the **Supplement**). The second was the protocol of the Chinese Hypertension League (CHL) guideline,[2] which recommended that BP estimate was the average of the first and second BP readings, if the absolute difference between these two BP readings was within 5 mmHg; or otherwise, was the average of all triplicate BP measurements (**eFigure 1B** in the **Supplement**). The third was the protocol of the European Society of Cardiology (ESC) guideline,[3] which proposed that the average of the second and third BP readings was taken as the BP estimate (**eFigure 1C** in the **Supplement**). The fourth was the protocol of the International Society of Hypertension (ISH),[4] which suggested that BP estimate could be the first BP reading if it was <130/85 mmHg; or otherwise, could be the average of the second and third BP readings (**eFigure 1D** in the **Supplement**). The fifth was the protocol of the Japanese Society of Hypertension (JSH) guideline,[5] which recommended that BP estimate was the average of two stable BP measurements, of which the absolute difference of BP readings should be within 5 mmHg; or otherwise, BP estimate was assumed

© 2020 Lin HJ et al. *JAMA Network Open.*

to be the average of the two consecutive BP readings with the smallest absolute difference, if absolute differences of the first two and the last two BP readings are both greater than 5 mmHg (**eFigure 1E** in the **Supplement**). The sixth was the protocol of the UK National Institute of Health and Care Excellence (NICE) guideline,[6] which indicated that, if the first BP reading <140/90 mmHg, BP estimate was simply the first BP reading; if the first BP ≥140/90 mmHg, BP estimate should be the lower one of the first and second BP readings if the absolute difference between these two BP readings was not significant, where the cutoff of 10 mmHg was used given that only 12% to 18% of BP measurement errors were greater than 10 mmHg;[7] or otherwise, the lower one of the second and third BP readings (**eFigure 1F** in the **Supplement**). Lastly, given the unpredictable short-term BP variability despite 5-minute rest and the occasional unparalleled variations between SBP and DBP, we proposed that the BP estimate was the average of any two BP measurements with the lowest SBP readings of the triplicate measurements. We dubbed it the Averaging the Lowest Two (ALT) protocol (**eFigure 1G** in the **Supplement**).

Statistical analysis

We categorized the "don't know" item in the questionnaire (**eTable 1** in the **Supplement**) as missing data. The habits of alcohol consumption were grouped into "No" if "Never/rarely" was answered, and into "Yes" if otherwise. The habit of the home BP monitoring was

© 2020 Lin HJ et al. *JAMA Network Open.*

classified as “Never” if the participants did not take any BP measurements 12 months before joining the campaigns. Hypertension was defined as one of the following was present: the mean BP ≥140/90, current use of anti-hypertensive medications, or prior medical diagnosis.

To compare intra-individual differences between BP estimates, complete records of triplicate BP readings were needed to calculate BP estimates using different BP estimation protocols. Incomplete records of triplicate BP measurements were mainly owing to patients’ preference and logistic limitations. we explored potential selection bias by performing inverse probability weighting-adjusted comparisons of the SBP and DBP averages between individuals with and without complete records of triplicate BP readings. The probability weighting was estimated using propensity scores, which included clinical features of age, sex, and body mass index; medical history of diabetes mellitus, coronary heart disease, stroke, and hypertension; lifestyle habits of smoking and alcohol consumption; and frequency of practicing home blood pressure monitoring in the preceding year.

A multivariable logistic regression model, where the averages of the triplicate SBP and DBP were adjusted, was constructed to explore whether the clinical features and BP variability patterns were related to the discrepant BP classifications among the seven BP estimates. The regression models included the variables of age (<50.0, 50.0 to 59.9, 60.0 to

© 2020 Lin HJ et al. *JAMA Network Open.*

69.9, and ≥70.0 years); body mass index (<24.0, 24.0 to 26.9, ≥27 kg/m^2); sex (male/female); medical history of coronary artery disease (yes/no), diabetes mellitus (yes/no), and hypertension (yes/no); current smoker (yes/no); alcohol consumption (yes/no); home BP monitoring (never, <1 day/week, 1 to 3 days/week, 4 to 6 days/week, daily); arm of BP measurement (right/left); and BP variability patterns (descending, fluctuating, ascending).

© 2020 Lin HJ et al. *JAMA Network Open.*

eFigure. Flowcharts Depicting the Blood Pressure Estimation Protocols

BP_1, BP_2, and BP_3 denote the first, second, and third BP readings, while SBP_1, SBP_2, and SBP_3 denote the corresponding systolic readings, respectively.

(A) American College of Cardiology (ACC) BP estimation protocol

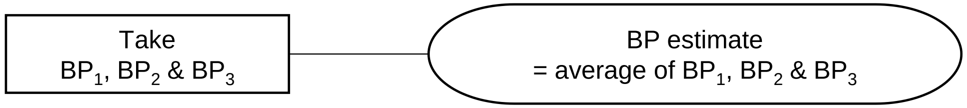

© 2020 Lin HJ et al. *JAMA Network Open.*

(B) Chinese Hypertension League BP (CHL) estimation protocol

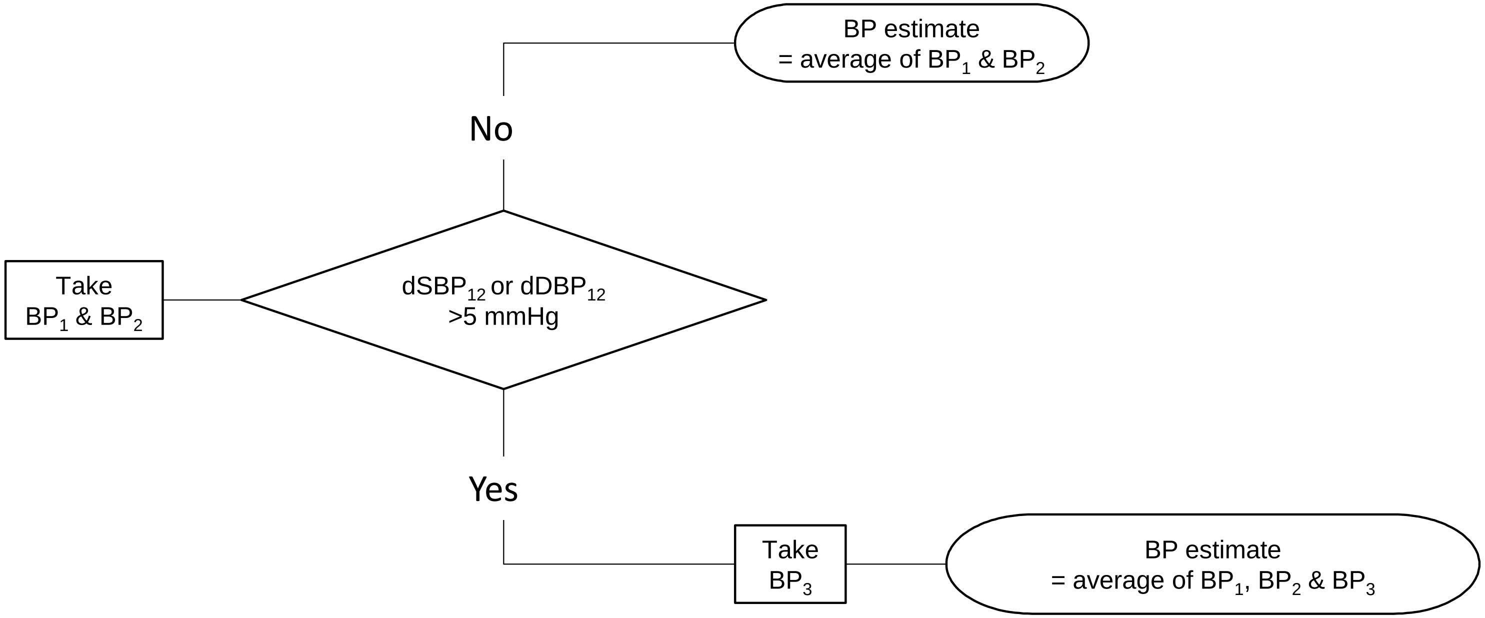

© 2020 Lin HJ et al. *JAMA Network Open.*

(C) European Society of Cardiology (ESC) BP estimation protocol

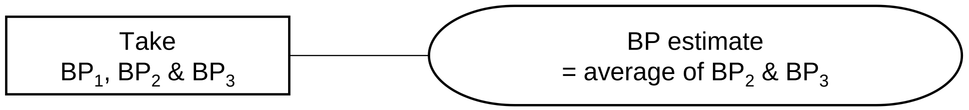

© 2020 Lin HJ et al. *JAMA Network Open.*

(D) International Society of Hypertension (ISH) BP estimation protocol

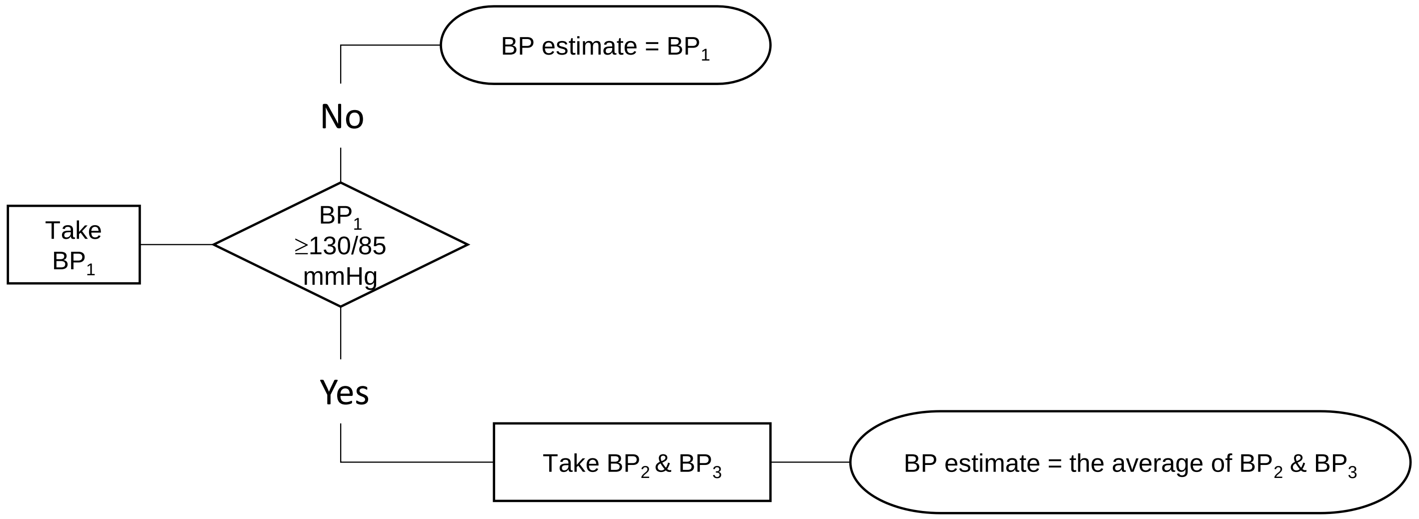

© 2020 Lin HJ et al. *JAMA Network Open.*

(E) Japanese Society of Hypertension (JSH) BP estimation protocol

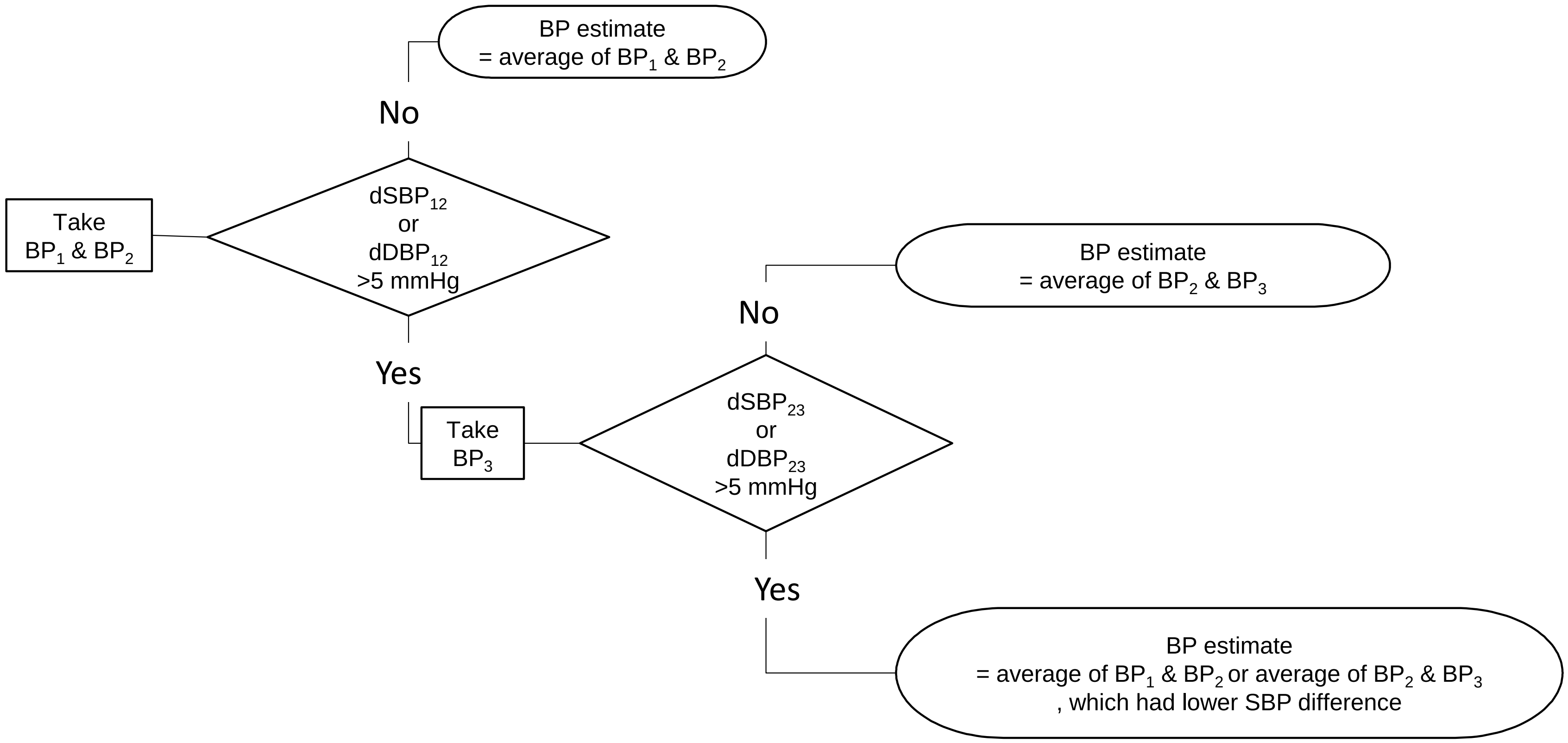

© 2020 Lin HJ et al. *JAMA Network Open.*

(F) National Institute of Health and Care Excellence (NICE) BP estimation protocol

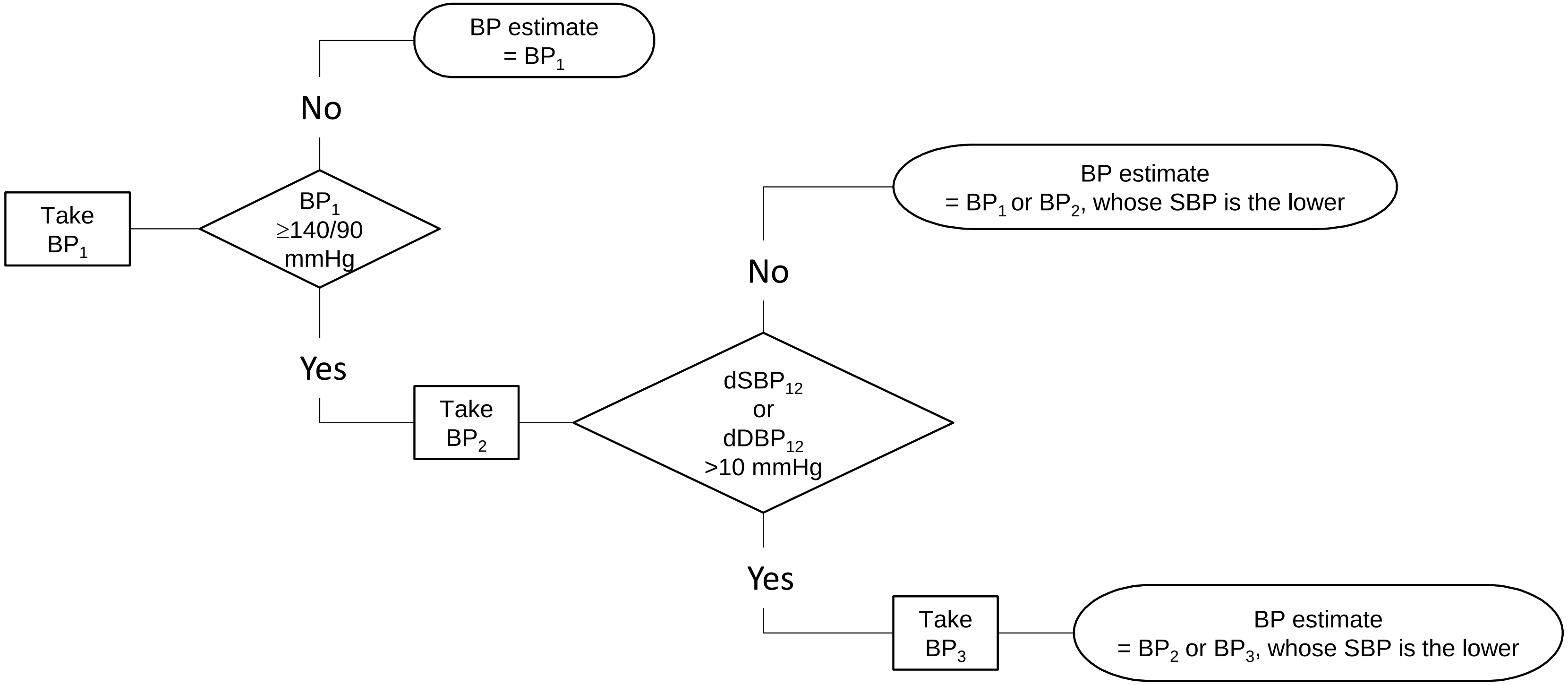

© 2020 Lin HJ et al. *JAMA Network Open.*

(G) "Averaging the Lowest Two" (ALT) BP estimation protocol

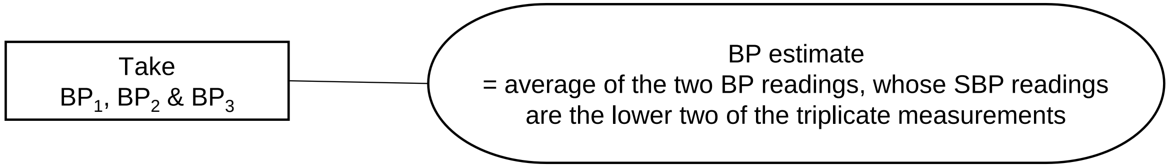

© 2020 Lin HJ et al. *JAMA Network Open.*

eTable 1. Questionnaire Used for Collecting Clinical Features, Lifestyle Habits, and Blood Pressure Records

□ **You understand the purpose of the blood pressure measurement campaign, and agree the contents of the questionnaire will be collected in an anonymous way and be used in hypertension studies.**

1. Sex	
2. Age (years)	
3. Body height (cm)	
4. Body weight (kgs)	

Medical history and lifestyle habits

1. Have you had your blood pressure measured in the last 12 months?	**Never**			
	**Everyday**	**4-6 days/wk**	**1-3 days/wk**	**<1 day/wk**
2. Have you ever been diagnosed with high blood pressure by a health professional	**No**	**Yes**		
3. Are you currently taking prescribed medication to treat high blood pressure?	**No**	**Yes**	**Don't know**	
4. Diabetes	**No**	**Yes**	**Don't know**	
5. Heart attack	**No**	**Yes**	**Don't know**	
6. Stroke	**No**	**Yes**	**Don't know**	
7. Current use of tobacco	**No**	**Yes**		
8. Alcohol consumption	**Never/rarely**	**1-3 times/mo**	**≥1 time/wk**	

BP measurement

1. Room temperature		℃
2. Date		
3. Time		
Arm of BP measurement	**Left**	**Right**

	**SBP**	**DBP**	**PR**
First			
Second			
Third			

© 2020 Lin HJ et al. *JAMA Network Open.*

eTable 2. Baseline Characteristics of Participants With and Without Complete Records of Triplicate BP Readings

	Triplicate BP readings		
	Complete	Incomplete	
	(n=62,647)	(n=18,394)	p
Age, years	57.1 (15.9)	56.5 (16.6)	<0.001
<50.0 years, n (%)	22,376 (35.7)	6,682 (36.3)	
50.0 to 59.9 years, n (%)	11,574 (18.5)	3,176 (17.3)	
60.0 to 69.9 years, n (%)	15,127 (24.1)	4,420 (24.0)	
≥70 years, n (%)	13,570 (21.7)	4,116 (22.4)	
Body mass index, kg/m^2	24.4 (3.7)	24.0 (3.6)	<0.001
<24.0 kg/m2, n (%)	31,232 (49.9)	10,046 (54.6)	
24.0 to 26.9 kg/m2, n (%)	18,182 (29.0)	4,997 (27.2)	
≥27.0 kg/m2, n (%)	13,233 (21.1)	3,351 (18.2)	
Woman, n (%)	31,922 (51.5)	9,607 (53.6)	<0.001
Medical history			
Stroke, n (%)	1,765 (2.9)	561 (3.2)	0.036
Coronary artery disease, n (%)	6,436 (10.4)	1,559 (8.9)	<0.001
Diabetes mellitus, n (%)	12,964 (21.2)	3,721 (21.0)	0.57
Hypertension, n (%)	28,096 (44.9)	8,774 (58.6)	<0.001
Lifestyle habits			
Current smoker, n (%)	10,818 (17.4)	3,087 (17.2)	0.42
Alcohol consumption, n (%)	7,904 (12.8)	2,451 (13.8)	0.001
Frequency of HBP monitoring, n (%)			<0.001
Never	18,086 (30.6)	6,481 (36.8)	
<1 day per week	22,447 (37.9)	5,352 (30.4)	
1 to 3 days per week	9,590 (16.2)	2,971 (16.9)	
4 to 6 days per week	3,394 (5.7)	1,199 (6.8)	
Daily	5,644 (9.6)	1,613 (9.2)	
The average of non-missing readings			
SBP	126.9 (16.8)	126.2 (18.1)	<0.001
DBP	78.0 (11.3)	78.2 (12.2)	0.066

For continuous variables, values are presented as mean (SD); and for categorical ones, are as number (percentage of non-missing data).

© 2020 Lin HJ et al. *JAMA Network Open.*

Abbreviations: BP, blood pressure; DBP, diastolic blood pressure; HBP, home blood pressure; SBP, systolic blood pressure.
*: P values were derived from comparing variables between individuals with and without complete records of triplicate BP readings.

© 2020 Lin HJ et al. *JAMA Network Open.*

eTable 3. Comparison of the Absolute Differences Between Systolic Blood Pressure and Diastolic Blood Pressure Readings of the Triplicate Measurements Compared With Pairs of the Lowest 2 Systolic Blood Pressure Readings

	The lower two SBPs of triplicate BP measurements					
	SBP_1 and SBP_2		SBP_2 and SBP_3		SBP_1 and SBP_3	
	(n=11,803)	p*	(n=31,359)	p*	(n=14,321)	p*
Absolute difference between SBP readings		<0.001		<0.001		<0.001
$dSBP_{12}$	3.9 (4.2)		6.1 (6.1)		5.6 (5.5)	
$dSBP_{23}$	5.9 (6.0)		3.5 (3.9)		5.9 (5.5)	
$dSBP_{13}$	6.0 (6.1)		7.5 (6.7)		3.7 (4.3)	
Absolute difference between DBP readings		<0.001		<0.001		<0.001
$dDBP_{12}$	3.5 (3.8)		3.8 (4.5)		3.8 (4.4)	
$dDBP_{23}$	3.6 (4.0)		2.9 (3.3)		3.7 (4.3)	
$dDBP_{13}$	4.1 (4.5)		4.6 (4.8)		3.6 (4.0)	

Abbreviations: BP, blood pressure; DBP, diastolic blood pressure; $dDBP_{12}$, absolute difference between the first and the second diastolic blood pressures; $dDBP_{23}$, absolute difference between the second and the third diastolic blood pressures; and $dDBP_{13}$, absolute difference between the first and the third diastolic blood pressures; $dSBP_{12}$, absolute difference between the first and the second systolic blood pressures; $dSBP_{23}$, absolute difference between the first and the second systolic blood pressures; and $dSBP_{13}$, absolute difference between the first and the third diastolic blood pressures; SBP: systolic blood pressure; SBP_1, the first systolic blood pressure; SBP_2, the second systolic blood pressure; SBP_3, the third systolic blood pressure.

*: p values indicate overall comparisons among the in-between absolute differences of SBP or DBP readings

© 2020 Lin HJ et al. *JAMA Network Open.*

eTable 4. Clinical Features and Blood Pressure Measures Among Participants Stratified by Variability Patterns of Triplicate Blood Pressure Measurements

	BP variability patterns of triplicate measurements			
	Descending	Fluctuating	Ascending	
	(n=18,363)	(n=38,892)	(n=5,392)	p
Age, years	57.5 (15.7)	57.0 (16.0)	57.1 (16.2)	0.002
Body mass index, kg/m2	24.5 (3.7)	24.3 (3.7)	24.3 (3.6)	<0.001
Woman, %	9,261 (51.0)	19,918 (51.7)	2,743 (51.4)	0.24
Medical history				
Stroke, %	533 (2.9)	1,067 (2.8)	165 (3.1)	0.30
Coronary artery disease, %	2,118 (11.7)	3,745 (9.8)	573 (10.8)	<0.001
Diabetes mellitus, %	4,019 (77.4)	7,817 (20.6)	1,128 (21.4)	<0.001
Hypertension, %	8,910 (48.5)	16,893 (43.4)	2,293 (42.5)	<0.001
Current smoker, %	3,254 (17.9)	6,625 (17.2)	939 (17.6)	0.12
Alcohol consumption, %	2,466 (13.6)	4,764 (12.5)	674 (12.7)	<0.001
Frequency of HBP monitoring, %				<0.001
Never	5,016 (28.9)	11,424 (31.1)	1,646 (32.5)	
<1 day per week	6,648 (38.3)	13,979 (38.1)	1,820 (35.9)	
1 to 3 days per week	2,880 (16.6)	5,890 (16.0)	820 (16.2)	
4 to 6 days per week	1,052 (6.1)	2,035 (5.5)	307 (6.1)	
Daily	1,756 (10.1)	3,409 (9.3)	479 (9.4)	
The arm of taking BP measurements				<0.001

© 2020 Lin HJ et al. *JAMA Network Open.*

Right arm, %		7,244 (43.0)	14,400 (40.4)	2,038 (41.9)	
Left arm, %		9,617 (57.0)	21,287 (59.6)	2,827 (58.1)	
Triplicate BP measurements					
SBP reading, mmHg					
	The first	134.1 (17.3)	126.8 (17.5)	119.9 (17.3)	<0.001
	The second	129.1 (16.5)	125.7 (17.4)	124.3 (17.1)	<0.001
	The third	124.9 (16.5)	125.6 (17.0)	128.4 (17.5)	<0.001
DBP reading, mmHg					
	The first	81.2 (11.8)	78.2 (12.1)	75.7 (12.3)	<0.001
	The second	78.9 (11.2)	77.6 (11.8)	76.8 (12.0)	<0.001
	The third	77.2 (10.9)	77.3 (11.6)	77.9 (12.0)	0.001
Pulse rate, beats per minute					
	The first	78.1 (10.7)	77.2 (10.9)	76.7 (11.1)	<0.001
	The second	76.6 (10.1)	76.6 (10.5)	76.9 (10.8)	0.22
	The third	75.7 (9.8)	76.4 (10.3)	77.1 (10.7)	<0.001
BP estimates derived from the BP estimation protocol					
SBP reading, mmHg					
	ACC protocol	129.3 (16.5)	126.1 (16.8)	124.2 (17.0)	<0.001
	CHL protocol	130.3 (16.4)	126.2 (16.9)	123.1 (17.0)	<0.001

© 2020 Lin HJ et al. *JAMA Network Open.*

		ESC protocol	127.0 (16.4)	125.7 (16.9)	126.3 (17.1)	<0.001
		ISH protocol	129.0 (14.5)	125.7 (16.7)	122.0 (19.5)	<0.001
		JSH protocol	129.2 (16.7)	125.9 (17.0)	124.0 (17.3)	<0.001
		NICE protocol	131.0 (14.9)	125.5 (16.3)	120.1 (17.6)	<0.001
		ALT protocol	127.0 (16.4)	124.3 (16.8)	122.1 (17.0)	<0.001
	DBP reading, mmHg					
		ACC protocol	79.1 (10.9)	77.7 (11.4)	76.8 (11.7)	<0.001
		CHL protocol	79.6 (11.0)	77.8 (11.5)	76.5 (11.8)	<0.001
		ESC protocol	78.0 (10.8)	77.4 (11.4)	77.4 (11.8)	<0.001
		ISH protocol	78.8 (10.3)	77.4 (11.4)	75.9 (12.6)	<0.001
		JSH protocol	79.1 (11.1)	77.6 (11.5)	76.7 (11.9)	<0.001
		NICE protocol	79.7 (10.6)	77.6 (11.5)	75.7 (12.3)	<0.001
		ALT protocol	78.0 (10.8)	77.1 (11.4)	76.2 (11.9)	<0.001

For continuous variables, values are presented as mean (SD); and for categorical ones, are as number (percentage of non-missing data).

Abbreviations: ACC, American College of Cardiology; BP, blood pressure; ALT: Averaging the Lowest Two; CHL, Chinese Hypertension League; DBP, diastolic blood pressure; $dSBP_{12}$: the absolute difference between the first and the second systolic blood pressure readings; $dSBP_{23}$: the absolute difference between the second and the third systolic blood pressure readings; ESC, European Society of Cardiology; HBP, home blood pressure; JSH, Japanese Society of Hypertension; NICE, National Institute of Health and Care Excellence; SBP, systolic blood pressure.

*: P values were derived from comparing variables among the three BP variability patterns.

© 2020 Lin HJ et al. *JAMA Network Open.*

eReferences

1. Whelton PK, Carey RM, Aronow WS, et al. 2017 ACC/AHA/AAPA/ABC/ACPM/AGS/APhA/ASH/ASPC/NMA/PCNA Guideline for the Prevention, Detection, Evaluation, and Management of High Blood Pressure in Adults: A Report of the American College of Cardiology/American Heart Association Task Force on Clinical Practice Guidelines. *Hypertension.* 2018;71(6):e13-e115.
2. Joint Committee for Guideline R. 2018 Chinese Guidelines for Prevention and Treatment of Hypertension-A report of the Revision Committee of Chinese Guidelines for Prevention and Treatment of Hypertension. *J Geriatr Cardiol.* 2019;16(3):182-241.
3. Williams B, Mancia G, Spiering W, et al. 2018 ESC/ESH Guidelines for the management of arterial hypertension. *Eur Heart J.* 2018;39(33):3021-3104.
4. Unger T, Borghi C, Charchar F, et al. 2020 International Society of Hypertension global hypertension practice guidelines. *J Hypertens.* 2020;38(6):982-1004.
5. Umemura S, Arima H, Arima S, et al. The Japanese Society of Hypertension Guidelines for the Management of Hypertension (JSH 2019). *Hypertens Res.* 2019;42(9):1235-1481.
6. National Institute for Health and Care Excellence. Hypertension in adults: diagnosis and management. NICE Guideline. https://www.nice.org.uk/guidance/ng136.

© 2020 Lin HJ et al. *JAMA Network Open.*

Published 28 August, 2019. Accessed November, 2019.

7. Stergiou GS, Alpert B, Mieke S, et al. A Universal Standard for the Validation of Blood Pressure Measuring Devices: Association for the Advancement of Medical Instrumentation/European Society of Hypertension/International Organization for Standardization (AAMI/ESH/ISO) Collaboration Statement. *Hypertension.* 2018;71(3):368-374.

© 2020 Lin HJ et al. *JAMA Network Open.*
